# Adrenocortical carcinoma presenting as varicocele and renal vein thrombosis: a case report

**DOI:** 10.1186/1752-1947-5-337

**Published:** 2011-08-01

**Authors:** Wisit Cheungpasitporn, John M Horne, Charles B Howarth

**Affiliations:** 1 Department of Internal Medicine, Bassett Medical Center, Cooperstown, NY 13326, USA

## Abstract

**Introduction:**

Adrenocortical carcinomas are rare aggressive tumors. Their annual incidence is approximately one to two per million among the population of the United States of America. Patients with active endocrine tumors often present with Cushing's syndrome accompanied by virilizing features. Conversely, patients with non-functioning tumors may present with symptoms related to a mass-occupying lesion, such as abdominal pain and flank pain. Although varicoceles and acute kidney injuries are common problems in medicine, they are uncommon presentations of these rare tumors and easy to miss. We report a case of a large adrenocortical carcinoma that presented as testicular pain, varicocele, and acute kidney injury secondary to renal vein thrombosis.

**Case presentation:**

A 54-year-old Caucasian man with a left-sided varicocele presented to our emergency department with lower abdominal pain and a decrease in urination. Four months previously, he had noticed pain and swelling in his left groin and had been diagnosed with left-sided varicocele. For one week, he began developing left-sided abdominal pain and decreased urination frequency, so he came to our emergency department for evaluation. His physical examination revealed a hard mass occupying the entire left side of his abdomen, crossing the midline, and extending to the pelvic brim. His blood tests showed acute kidney injury and mild anemia. Computed tomography of his abdomen showed a large retroperitoneal mass on the left side, displacing the left kidney inferiorly and the spleen superiorly with thoracic epidural compression. Thrombus was also identified in his left renal vein and inferior vena cava. Computed tomography of his chest showed bilateral pulmonary nodules. A computed tomography-guided abdominal mass biopsy was performed, and the diagnosis of adrenocortical carcinoma was made on the basis of pathology and immunohistochemistry. His hormonal evaluations were normal. His kidney function improved with intravenous hydration and anti-coagulation treatment. Unfortunately, the adrenal mass was unresectable because of the extent of the tumor. Treatment with mitotane, an adrenocorticolytic drug, was started with concomitant with irradiation of a lesion at T5, followed by combination chemotherapy thereafter.

**Conclusion:**

Unilateral right-sided varicoceles are rare and should alert the clinician to possible underlying pathology causing inferior vena caval obstruction. Left-sided varicoceles, in contrast, are common secondary to the venous anatomy of the left testis; however, the enlargement of the left testicle can be associated with blockage of the left testicular vein by tumor invasion of the left renal vein. Varicoceles could be an early presentation of a non-functioning adrenocortical carcinoma. Acute kidney injury can occur as a result of mass effect or thrombosis of renal vessels. Large tumors can cause abdominal pain as a late manifestation. Physicians should perform a complete abdominal examination in every patient with varicocele or testicular pain.

## Introduction

Adrenocortical carcinomas (ACCs) are rare aggressive tumors; their annual incidence is approximately 1 to 2 per million among the population of the United States of America [1]. Approximately 60% of ACCs are functional tumors [2]. Patients can present with Cushing's syndrome alone (45%), a mixed Cushing's and virilization syndrome (25%), or virilization alone (< 10%) [3]. Conversely, patients with non-functioning tumors more commonly present with an enlarging abdominal mass and abdominal or back pain or with an incidental finding on radiographic imaging called an "adrenal incidentaloma." Although varicocele and acute kidney injury are common conditions in medicine, they are uncommon presentations of these rare tumors. We report a case of a patient with a large ACC that presented as testicular pain, varicocele, and acute kidney injury secondary to renal vein thrombosis.

## Case presentation

**Figure 1:**
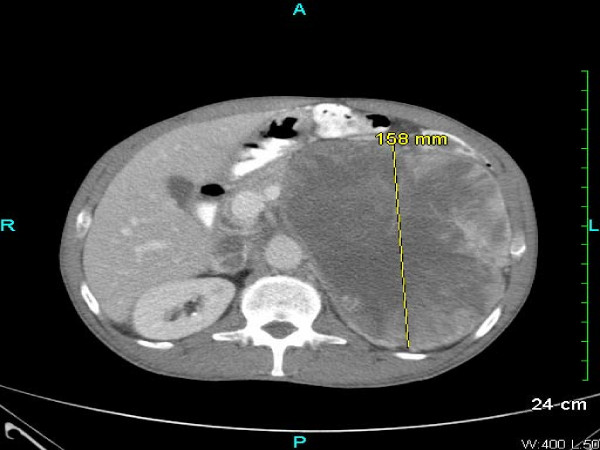
**Abdominal computed tomography (CT) examination of the patient**. This abdominal CT image shows a large left-sided retroperitoneal mass.

**Figure 2:**
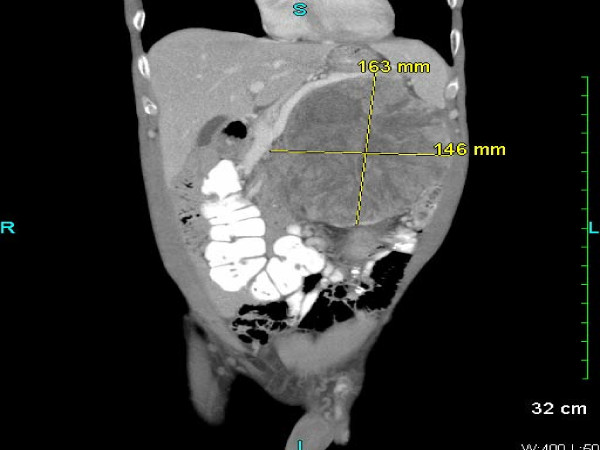
**Sagittal view of abdominal computed tomography (CT) examination of the patient**. This abdominal CT scan shows a large retroperitoneal mass displacing the left kidney inferiorly and the spleen superiorly.

**Figure 3:**
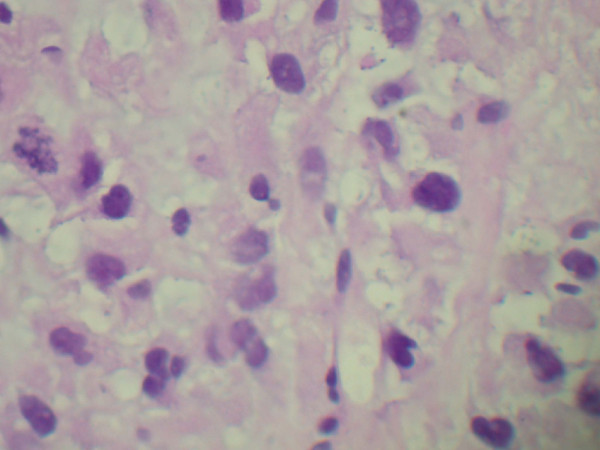
**Histopathology of a tissue biopsy specimen taken from the patient**. This slide shows a mitotic figure in the middle of the left-hand edge of the field.

**Figure 4:**
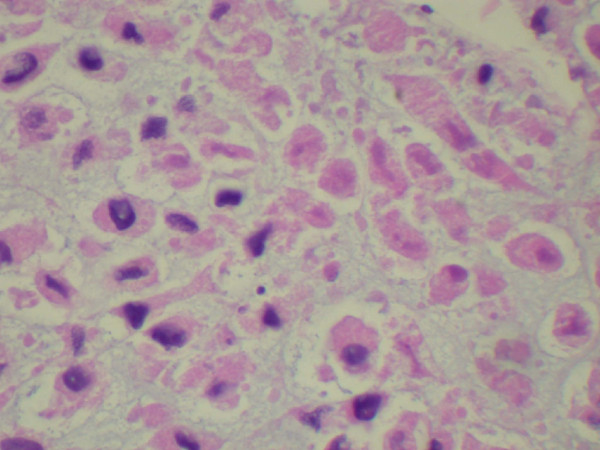
**Histopathology of a tissue biopsy specimen taken from the patient**. This slide shows tissue necrosis.

A 54-year-old Caucasian man with a history of ischemic stroke and ischemic cardiomyopathy presented to our emergency department with constant left-sided lower abdominal pain and decrease in urination of one week's duration. Four months prior to presentation, he had noticed pain and swelling in his left groin. Because of his concerns of a hernia, he sought clinical evaluation. His family physician sent him for an ultrasound of the scrotum, which revealed a left-sided varicocele. He was then referred to a urologist. For one week, he developed continuous, unrelenting left-sided abdominal pain localized primarily in the left lower quadrant. He had diminished appetite and noted a 12-pound weight loss during the prior one month. Because of decreased urinary frequency, he came to our emergency department for evaluation. His family history was negative for malignancy. His physical examination revealed a hard mass occupying the entire left abdomen, crossing the midline, and extending to the pelvic brim, as well as the presence of a left-sided varicocele. He had no lymphadenopathy or hepatomegaly and no clinical signs of hormone access of 1.7 from a baseline of 1.0, hyponatremia (serum sodium 130 mmol/L), and normocytic anemia (hemoglobin 10.9 g/dL, hematocrit 34.5%, mean corpuscular volume 82.5 fL, and mean corpuscular hemoglobin 26.1 pg.) His hormonal evaluations, including fasting blood glucose, serum potassium, adrenocorticotropic hormone (ACTH), morning serum cortisol, and androgen levels, were normal. Renal ultrasound showed that the left kidney was inferiorly displaced by what was thought to be an enlarged spleen. His home medication of lisinopril was discontinued. Intravenous fluid was started. Contrast-enhanced computed tomography (CT) of the abdomen and pelvis performed after the patient's renal function improved showed a large retroperitoneal mass on the left side displacing the left kidney inferiorly and the spleen superiorly with T5 epidural compression (Figures 1 and Figure 2). Thrombus was also identified in the left renal vein extending into the inferior vena cava. A CT chest scan showed bilateral pulmonary nodules compatible with metastasis. Anti-coagulation therapy, a 5 mg/day dose of warfarin adjusted according to International Normalized Ratio levels and five days of 1 mg/kg enoxaparin administered subcutaneously every 12 hours for bridging therapy were initiated because of thrombosis of the blood vessels. The patient's acute kidney injury improved after intravenous fluid and anti-coagulation treatment. During the course of his hospitalization, he was seen by our medical oncologist, who managed his anti-coagulation therapy and arranged for the biopsy. A CT-guided biopsy of the abdominal mass was performed. Immunohistochemistry showed malignant cells with abundant amounts of eosinophilic cytoplasm and a rosette pattern of the cells around his blood vessels. Occasional, very enlarged, bizarre nuclei were observed. Mitoses were rare but could be identified (Figures 3 and 4). Immunohistochemistry performed to detect primary adrenal origin was positive for calretinin, melan-A, vimentin, and synaptophysin. His Ki-67 proliferation index was 12%. This presentation was consistent with primary ACC. The diagnosis of ACC stage IV was made. Laboratory tests performed for hormornal evaluation, including fasting blood glucose, serum potassium, cortisol, and urinary metanephrine levels, were normal. A nuclear cardiac stress test was performed, which showed borderline anterior ischemia and mild to moderate systolic dysfunction with a left ventricular ejection fraction of 38%. Unfortunately, the adrenal mass was determined to be unresectable because of local unresectability and metastatic disease.

Mitotane, an adrenocorticolytic drug, was started at an initial dose of 1 g/day and the dose was later increased to maintain plasma levels between 14 μg/mL and 20 μg/mL, which was well tolerated. Irradiation of the lesion at T5 that was causing the epidural compression was followed by combination chemotherapy consisting of etoposide, doxorubicin, and cisplatin. The patient understood that the goal of therapy was to control his symptoms and hopefully to achieve better quality of life and prolonged survival.

## Discussion

Varicoceles most commonly present as unilateral dilatation of the pampiniform plexus of veins above the left testis. Left-sided varicoceles are present in approximately 10% to 20% of men and are believed to be secondary to the venous anatomy of the left testis. Right-sided varicoceles usually occur as bilateral processes and are apparent in 10% of clinical cases and in as many as 50% of subclinical cases. Unilateral right-sided varicoceles are very rare and should alert the clinician to possible underlying pathology causing inferior vena cava obstruction such as retroperitoneal malignancy [4]. On the other hand, left-sided varicoceles secondary to the venous anatomy of the left testis are very common. Enlargement of the left testicle can be associated with blockage of the left testicular vein by tumoral invasion of the left renal vein and should be evaluated for the presence of retroperitoneal malignancy as well.

The most common retroperitoneal malignancy causing this presentation is right-sided renal cell carcinoma. Several other tumors have been mentioned as the cause of right-sided varicocele, such as Burkitt's lymphoma [5] or Wilms tumor. An aortic pseudoaneurysm presenting as right-sided varicocele has also been reported. Renal vein thrombosis is fairly uncommon and may occur after trauma to the abdomen or the back or as a result of scar formation, stricture, or tumor formation, most commonly renal cell carcinoma. Also, renal vein thrombosis is frequent in ACC and is part of the European Network for the Study of Adrenal Tumours (ENSAT) staging system.

A MEDLINE search of the literature from 1966 to the present revealed no previous documentation of an ACC presenting as a right-sided varicocele or acute kidney injury secondary to renal vein thrombosis. Only one case report of ACC presenting as right-sided varicocele was found [6].

A hormonal work-up for functional ACCs is widely considered mandatory; however, the question whether to perform this evaluation in apparently asymptomatic patients has been debated. ENSAT recommends performing the following tests to determine the secretory activity of the tumor: levels of fasting blood glucose, serum potassium, cortisol, ACTH, 24-hour urinary free cortisol, fasting serum cortisol at 8 AM following a 1 mg dose of dexamethasone at bedtime, adrenal androgens (dehydroepiandrosterone sulfate, androstenedione, testosterone, and 17-OH progesterone), and serum estradiol in men and post-menopausal women [7]. Plasma metanephrine level or urinary metanephrine and catecholamine levels may be measured to exclude pheochromocytoma. Plasma aldosterone and renin levels may be measured in patients with hypertension and/or hypokalemia. CT scanning can usually distinguish adenomas from ACCs. The size of the adrenal mass visualized on imaging studies is the single most important criterion to help diagnose malignancy. In the series reported by Copeland [8], 92% of adrenal tumors were greater than 6 cm in diameter. Magnetic resonance imaging (MRI) is complementary to CT in that local invasion and involvement of the vena cava are more readily identifiable. A fine-needle aspiration biopsy cannot distinguish a benign adrenal mass from an adrenal carcinoma. However, it can distinguish an adrenal tumor from a metastatic tumor. Capsular or vascular invasion is the most reliable sign of cancer. In the absence of these findings, the Weiss histopathologic system [9,10] is the most commonly used method for assessing the likelihood of malignancy because of its simplicity and reliability. Immunohistochemistry is also helpful in rendering the diagnosis [8,11].

A variety of staging systems have been used for ACC. The Union for International Cancer Control (UICC) proposed the first TNM classification of Malignant Tumors for ACC in 2003. However, an analysis based on data from the German ACC Registry revealed several shortcomings of this classification system. Therefore, ENSAT developed a revised staging system. The superiority of the ENSAT staging system over the 2004 UICC/American Joint Committee on Cancer classification system for prognostication was confirmed in a recent North American study. Estimated five-year disease-specific survival rates of patients with stage I and stage IV cancer in the studies were 82% and 13%, respectively [12,13].

Nowadays, adrenocortical cancer is often diagnosed after a great delay, when the cancer is very advanced, as shown in the present case report. The only potentially curative treatment for ACC is surgical resection [14], which is technically possible in most patients with stages I to III disease. The most important predictor of survival in patients with adrenal cancer is the adequacy of resection. Patients who undergo complete resection have five-year actuarial survival rates ranging from 32% to 48%, whereas median survival is less than one year in patients who undergo incomplete excision. Other treatment options include treatment with mitotane, an adrenocorticolytic drug, as well as adjuvant chemotherapy and palliative irradiation [15].

## Conclusion

Testicular pain and varicocele could be an early presentation of non-functioning ACCs. Acute kidney injury can occur as a result of a mass effect or as a result of thrombosis of renal vessels. Large tumors can cause abdominal pain in the late manifestation and unresectable stage. The diagnosis of varicoceles necessitates evaluation of the abdomen and retroperitoneum for underlying malignancy. To our knowledge, herein we report the first case of a large, left-sided, non-functioning ACC presenting as a left-sided varicocele and acute kidney injury secondary to left renal vein thrombosis.

## Abbreviations

ACC: adrenocortical carcinoma; ACTH: adrenocorticotropic hormone; CT: computed tomography; ENSAT: European Network for the Study of Adrenal Tumours; UICC: Union for International Cancer Control.

## Consent

Written informed consent was obtained from the patient for publication of this case report and any accompanying images. A copy of the written consent is available for review by the Editor-in-Chief of this journal.

## Competing interests

The authors declare that they have no competing interests.

## Authors' contributions

WC, JMH, and CBH were involved in the diagnosis and treatment of the patient. WC drafted the manuscript. JMH and CBH revised the manuscript. All authors read and approved the final manuscript.
